# Hepatitis B, C and D virus prevalence in children and adults in Mbeya Region, Tanzania: results from a cohort study 2002 - 2009

**DOI:** 10.11604/pamj.2021.39.174.26553

**Published:** 2021-07-06

**Authors:** Guenter Froeschl, Michael Hoelscher, Lucas Henze Maganga, Inge Kroidl, Petra Clowes, Steffen Geis, Elmar Saathoff, Dieter Hoffmann, Ulrike Protzer, Arne Kroidl

**Affiliations:** 1 Division of Infectious Diseases and Tropical Medicine, University Hospital, Ludwig-Maximilians-Universität of Munich, Munich, Germany,; 2 German Centre for Infection Research (DZIF), Partner Site Munich, Munich, Germany,; 3 National Institute for Medical Research, Mbeya Medical Research Center, Mbeya, Tanzania,; 4 Helmholtz Zentrum München/Technische Universität München, Institute of Virology, Munich, Germany

**Keywords:** Hepatitis B, hepatitis C, hepatitis D, HIV, adult, pediatric, Tanzania

## Abstract

**Introduction:**

sub-Saharan Africa bears a high prevalence for hepatitis B virus (HBV) infection. This analysis aims at elucidating the exposure to HBV across different age groups in Mbeya Region in Tanzania and determines prevalences of hepatitis C (HCV) and hepatitis delta antigen (HDV) infections.

**Methods:**

plasma samples from children and adults with defined HIV status were analysed for HBV, HCV and HDV markers.\

**Results:**

hepatitis B (HBs)-antigen positivity was 8.3% (3/36) in the 0 to 5 years age group, 13.3% (8/60) in the 6 to 7 years, 17.2% (10/58) in the 8 to 14 years and 13.3% (8/60) in the 15 to 18 years age groups. In adults 5.0% of samples were HBs-antigen positive. Overall, 17.1% were HIV-1 positive. Adults infected with HIV-1 were significantly more often HBs-antigen positive (7.5%) than HIV-1 negative adults (4.5%; p<0.05). A serological sub-study including 174 adults showed that both total anti-HBs and total anti-HBc positivity increased with age in HBs-antigen negative participants. Across all age groups, HCV antibodies were found in 9 individuals, HDV antibodies in 3 individuals.

**Conclusion:**

children presented a high prevalence of HBs-antigen carriers, with lower levels in the younger children. Among adults, the overall prevalence of HBs-antigen was lower than in children, either corresponding to clearance of HBV over time or due to a die-off effect. HBs-antigen positive adults had higher frequencies of anti-HBc- and anti-HBe-antibodies, indicating better immunological control of HBV infection than children. This supports claims that HBV infections in Africa are mostly acquired in childhood and to a large extent cleared again by adulthood. One in 20 adults remains chronically infected, emphasising the importance of HBV vaccination strategies.

## Introduction

According to a World Health Organization report from 2017, 257 million individuals are assumed to be chronically infected with hepatitis B virus (HBV) and about 884,000 die as a result of the infection [1]. At the same time, 37 million are globally infected with HIV, with an estimated 1.1 million deaths per year [2]. The number of HBV and HIV co-infection is estimated globally at 2.7 million [1]. Sub-Saharan Africa hosts both a relatively high prevalence in the general population for HBV at an estimated 8.8% and for HIV at 2.5% [2,3]. Both infectious diseases share routes of infection and thus have comparable individual risk profiles [4]. As a result, the populations infected with either one of the two chronic viral infections have a higher risk of co-infection with the respective other virus than the general population [5,6]. At the same time both infections do interact and lead to a worsening of the natural course of infection, both in HBV and HIV [7-9].

Immunocompetent adults are assumed to clear an acute HBV-infection in about 95% of cases, whereas children are reported to suffer more often from chronic courses [10,11]. In Asia perinatal HBV transmission has been associated as the predominant cause of infant HBV-infection [12], however, vertical transmission seems to be less important in Africa [13-15]. In Africa, most HBV transmission have been reported to occur before the age of five years through close contact within households, medical procedures or traditional scarification [16-18].

The United Republic of Tanzania is located in East Africa, with a population of 49 million and a life expectancy at birth of 61 years [19]. The Tanzania HIV/AIDS and malaria indicator survey 2007-08 gives an estimate for the HIV prevalence in Mbeya Region of 9.2% in the studied population aged 15 to 49 years [20]. A systematic review by Schweitzer *et al*. in the Lancet from 2015 indicates an HBV prevalence of around 7% [3]. HIV treatment programmes have reached considerable coverage in the country in the 2000´s and in the same period vaccination programmes against HBV have been initiated for children. According to WHO data, the coverage for 3-dose HBV vaccine administration in Tanzania is given in a range between 83% and 95% in the time period 2002 to 2010 [21]. However, detailed knowledge on prevalence of HBV infections or rates of mother-to-child transmissions are only available on a patchy basis [22]. In addition, information on the regional coverage of the HBV vaccination campaigns is not available. A study in 348 health care workers as vulnerable target population in Tanzania in 2015 has shown a coverage as little as 33.6% [23]. This study aimed at generating evidence on the prevalence of HBV in the light of HIV status in a cohort of children and adults in the Mbeya Region in Tanzania. In addition, data on HCV and HDV prevalence are presented. The results are expected to support policy makers in allocation of resources towards prevention and treatment of both HIV and HBV in Tanzania.

## Methods

**Figure 1:**
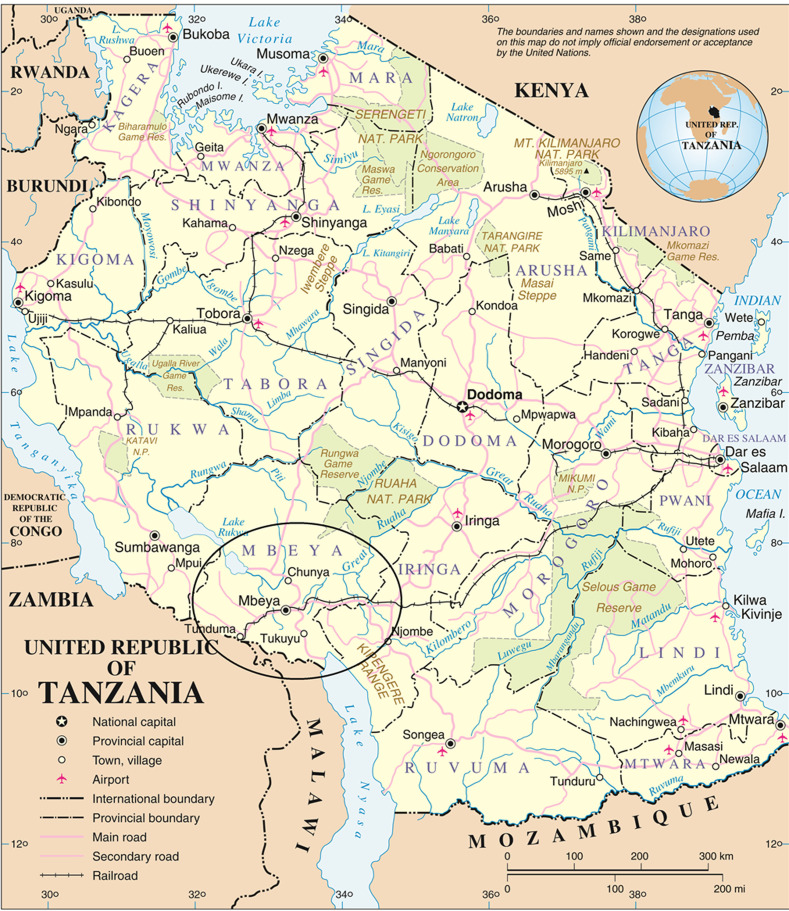
map of Tanzania based on the United Nations Cartographic Section, Map No. 3667 Rev.6, January 2006; written permission has been given by the United Nations Cartographic Section to use and modify the map

**Study setting and design:** the Mbeya Region is an administrative entity in the Southwest of Tanzania with a surface of 60,000 km^2^and a population of 2.7 million. The region is characterized by rural communities, with agriculture being the major source of income [24] (Figure 1). The Tanzanian National Institute of Medical Research - Mbeya Medical Research Center (NIMR-MMRC) was established in the city of Mbeya with the aim of investigating infectious diseases that are of public health concern for the region, with an initial focus on HIV. Several larger cohorts have been continuously maintained for this purpose. In this study we combined the cross-sectional surveillance results for prevalence of HIV infection of a large paediatric cohort within the EMINI study, the cross-sectional surveillance results for prevalence of HBV and HIV infection of a large adult cohort from the CODE study, and the results of a serological post-hoc analysis from a subset of stored blood samples collected throughout these two large cohort studies conducted in the Mbeya region between 2002 and 2011.

**Study population:** the EMINI cohort (Establishment of the infrastructure to Evaluate and Monitor the Impact of New Interventions) with participants from the general population in the Mbeya Region was established in 2005 with the intention to assess the epidemiology of infectious diseases and the impact that new interventions would have on these. The EMINI cohort included randomly selected 10% of all households in the study area. A total of more than 17,000 participants were longitudinally followed up over the course of 5 years [25-27]. The Cohort Development (CODE) cohort was initiated in 2002 in order to investigate strategies for recruitment of individuals for future vaccine trials. This cohort comprised more than 3,000 individuals [28]. The EMINI cohort contributed the paediatric participants to this study, the CODE cohort contributed the adult individuals.

**Data collection and laboratory analysis:** as part of the general study protocol of both EMINI and CODE cohorts, all included individuals were tested by rapid diagnostic tests for HIV. In addition, the participants of the CODE study, which constitute the adult subgroup in our analysis, were tested by rapid diagnostic tests for hepatitis B virus surface antigen (HBs antigen). Plasma samples of individuals of both EMINI and CODE studies were stored at -80°C. For the EMINI cohort the total population of approximately 42,000 households from nine distinct sites in Mbeya Region was georegistered using hand-held global positioning system (GPS) devices. A geographically stratified and then randomly selected subgroup of 10% (4,283) of the households were included in the EMINI cohort. The sociodemographic data provided in this study were age and sex. Data and samples for the EMINI study and used in this analysis were collected between July 2008 and May 2009; samples for the paediatric follow-up study were then collected in the time period June 2009 to May 2010. The paediatric cohort that has been drawn upon by this study comprises 9,368 individuals. It has to be noted that the upper limit of the age range is <19 years of age, therefore including individuals of 18 years of age. This results in some overlap of age ranges between the paediatric EMINI cohort and the adult CODE cohort.

For the serological and molecular marker study, a subset of plasma samples stored at -80°C were purposively selected by age, resulting in 60 individuals each for the age groups 0 to <6, 6 to <8, 8 to <15 and 15 to <19 years, respectively. Samples were also selected purposely by HIV status, to ensure a proportion of at least 20% of HIV positive individuals within each age group and to allow for adequately sized sub-groups with regard to HIV status. The selection of samples within age and HIV status groups was done by simple random sampling.

Samples of the adult CODE cohort used in this study were collected between September 2002 and April 2003. Of the 3,096 individuals who participated in CODE, 747 (24.1%) had to be excluded due to missing information on HIV status or HBV status, or due to a reported age at inclusion of less than 18 years, resulting in an eligible cohort of 2,349 (75.9%) individuals. The baseline visit for these individuals as part of the CODE study was used to determine hepatitis B and HIV prevalence for the adult cohort. A subset of plasma samples stored in -80°C, purposely selected by HBs-antigen status and HIV infection status, was further used for additional serological and molecular examinations. The selection of samples within HBs-antigen and HIV status subgroups was executed by mode of simple random sampling. This subset of 174/2,349 (7.4%) participants comprised 42 (24.1%) individuals that had tested positive for HBs-antigen; in total 79/174 (45.4%) individuals were HIV positive; nine out of 174 individuals (5.2%) were positive for both infectious diseases. However, it has to be kept in mind that these percentages are proportions that arise from the purposive sampling procedure and do not represent prevalences in the general population. Especially in younger paediatric age groups the volume of the blood samples was insufficient, leading to an exclusion of samples at this stage (24 for the age group 0 to <6; 2 for the age group 8 to <15). The remaining subsets of the paediatric EMINI cohort comprised 36 children aged 0 to <6, 60 children aged 6 to <8, 58 children aged 8 to <15 and 60 adolescents aged 15 to <19 years of age.

**Assays:** at the time of inclusion into the study cohorts, all participants were tested for HIV infection by applying a dual testing strategy with commercially available rapid diagnostic tests. The paediatric cohort of the EMINI study was tested with SD-Bioline HIV-1/2 3.0 (Standard Diagnostics, Kyonggi-do, South Korea); the adult cohort of the CODE study was tested with determine HIV-1/2 (Abbott Laboratories, Abbott Park, USA). The second test for both paediatric and adult cohorts was Enzygnost HIV-1/2 plus (Behring, Liederbach, Germany). Discordant results were confirmed in both paediatric and adult cohorts by the HIV blot 2.2 Western Blot (Genelabs/ Abbott, Wiesbaden, Germany). In addition, all adult individuals from the CODE study were tested for presence of HBs-antigen (MONOLISA HBsAg ULTRA, Bio-Rad, Hercules, USA) as part of the original CODE study protocol. Positive results were confirmed by the HBs-antigen neutralization test MONOLISA HBsAg ULTRA confirmatory (Bio-Rad, Hercules, USA). The serological markers HBs-antigen, anti-HBs-antibodies, anti-HBc-antibodies, anti-HBe-antibodies, HBe-antigen and anti-HCV-antibodies were detected by a chemiluminescent microparticle immunoassay (CMIA) by the Architect Immunoassay Analizer (Abbott Laboratories, Lake Bluff, Illinois, United States). Anti-HDV-antibodies were detected by an enzyme linked immunosorbent assay on the BEP III analyser (Siemens Healthcare, Erlangen, Germany). HBV DNA was quantitatively detected using an in-house polymerase chain reaction (PCR) assay.

**Statistical analysis:** age was recorded for the paediatric cohort in years and months as 1/12^th^ of one year. In the adult cohort age was recorded in full years. Results for all serological markers and for HBV DNA detection were recorded and analysed dichotomously in this study as positive or negative. Categorical variables were compared for significant correlations by using χ^2^test, or by using Fisher´s exact test for table cell sizes <5. All statistical analyses were performed using Stata/SE (StataCorp. 2015. Stata Statistical Software: Release 14. College Station, Texas, USA). Missing data occurred in the serological analyses in the paediatric subgroup due to small sample volumes. Here, observations with missing data were excluded, thus resulting in reduced denominators. This is indicated in the respective results sections.

**Ethical considerations:** this study follows the requirements of the Helsinki Declaration. All adult participants had signed an informed consent form prior to inclusion and for all minors below the age of 18 years their legal guardians signed the informed consent form. In addition and when appropriate, minors signed an assent form. The EMINI (opinion number NIMR/HQ/R.8a/Vol. IX/358) and the CODE (MRH/R.10/9) studies were approved by the Tanzanian National Institute of Medical Research review board.

**Funding:** the EMINI study was funded by the European Commission (SANTE/2004/078-545/130 & SANTE/2006/129-931). The funding agencies had no role in study design, data collection, data analysis, decision to publish, or preparation of the manuscript.

## Results

**Paediatric cohort:** the overall prevalence of HIV infection in the paediatric EMINI participants was 1.5% (142/9,368), the HIV prevalence by age group is given in Table 1. HBV data for the overall cohort was not available, therefore a meaningful correlation between HIV and HBV cannot be given for the paediatric cohort of this study. Of the paediatric cohort, 240 samples were selected for the paediatric serological marker sub-cohort. Here, the age profile is defined by the purposive selection of samples by age stratification, as detailed above in the methods section and is thus not representative for the underlying population. Twenty-four samples for the age group 0 to <6 years of age and 2 samples for the age group 8 to <15 years of age could not be processed due to containing too little volume, resulting in 214 samples for further serological analysis. Males represented 103/214 (48.1%) of this sub-cohort.

**Table 1:** HIV prevalence for the overall EMINI paediatric cohort (2009) and overall CODE adult cohort (2004) and hepatitis B surface antigen (HBsAg) by HIV status for the overall CODE adult cohort from the general population within the Mbeya Region in Tanzania

	Paediatric cohort (EMINI study)				Adult cohort (CODE study)		
Age groups (years)	0 to <6	6 to <8	8 to <15	15 to <19	18 to <25	25 to <30	≥30
HIV-infected	59/2,756 (2.1%)	20/1,222 (1.6%)	37/3,841 (1.0%)	26/1,549 (1.7%)	109/1,057 (10.3%)	97/431 (22.5%)	196/861 (22.8%)
HBsAg positive overall	ND	ND	ND	ND	51/1,057 (4.8%)	20/431 (4.6%)	47/861 (5.5%)
HBsAg positive among HIV-positive	ND	ND	ND	ND	4/109 (3.7%)	9/97 (9.3%)	17/196 (8.7%)
HBsAg positive among HIV negative	ND	ND	ND	ND	47/948 (5.0%)	11/334 (3.3%)	30/665 (4.5%)

Data is indicated in numbers (n/N; %); ND: not determined

**Figure 2:**
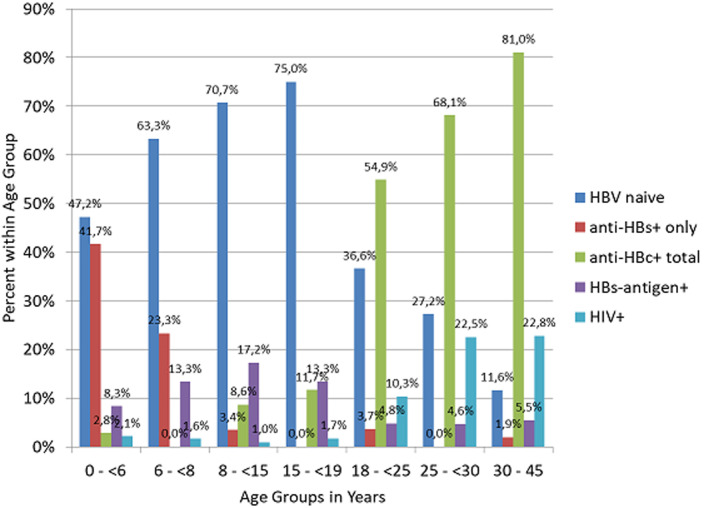
histogram on proportions of prevalences of seromarkers by age groups; bars indicate prevalences of serological constellations within the indicated age groups

**Hepatitis B virus:** HBs-antigen positivity was 8.3% (3/36) in children below 6 years, 13.3% (8/60) in the 6 to <8 years age group, 17.2% (10/58) in the 8 to <15 years age group and 13.3% (8/60) in the age 15 to <19 years age group (Table 1 and Figure 2). No significant correlation between age and prevalence of HBs-antigen positivity was found in the χ^2^test. In a pooled analysis in HBs-antigen negative children, an isolated anti-HBs-antibody positivity as indication of past anti-HBV vaccination was detected in 34.1% of the age group 0 to <8 years of age, compared to 2.0% in the age group 8 to <19 years. Conversely, anti-HBc-antibody positivity was detected in 1.2% of individuals in the age group 0 to <8 (0 to <6 and 6 to <8 combined) years compared to 12.0% in the age group 8 to <19 years (8 to <15 and 15 to <19 combined). Individuals serologically naive to HBV were found at a frequency of 64.7% in the age group 0 to <8 years, compared to 86.0% in the age group 8 to <19 years of age (Table 1). For all paediatric subgroups combined, the HBe status was determined. In HIV negative individuals there were 18 HBs-antigen positive cases, in which in 13 an anti-HBe assay could be performed; in HIV positive individuals there were 11 HBs-antigen positive cases, in which in 10 an anti-HBe assay could be performed. There was a higher prevalence of anti-HBe (3/13, 23.1%) in HIV negative individuals compared to HIV positive individuals (1/10, 10.0%, Table 2). However, this effect was not significant (Fisher´s exact p=0.18).

**Table 2:** hepatitis B, C and D outcomes for selected subsets from the EMINI paediatric cohort (2009)

	Paediatric cohort											
Age groups (years)	0 to <6			6 to <8			8 to <15			15 to <19		
	Overall N=36	HIV+ N=13	HIV- N=23	Overall N=60	HIV+ N=12	HIV- N=48	Overall N=58	HIV+ N=23	HIV- N=35	Overall N=60	HIV+ N=14	HIV- N=46
Male gender	17/36 (47.2%)	6/13 (46.2%)	11/23 (47.8%)	33/60 (55.0%)	6/12 (50.0%)	27/48 (56.3%)	26/58 (44.8%)	9/23 (39.1%)	17/35 (48.6%)	27/60 (45.0%)	4/14 (28.6%)	23/46 (50.0%)
HBsAg+	3/36 (8.3%)	3/13 (23.1%)	0/23 (0.0%)	8/60 (13.3%)	2/12 (16.7%)	6/48 (12.5%)	10/58 (17.2)	4/23 (17.4%)	6/35 (17.1%)	8/60 (13.3)	2/14 (14.3%)	6/46 (13.0%)
HBeAg+	2/3 (66.7%)	2/3 (66.7%)	0/0	*0/7 (0.0%)	0/2 (0.0%)	*0/5 (0.0%)	4/10 (40.0%)	2/4 (50.0%)	2/6 (33.3%)	*2/3 (66.7%)	*1/1 (100.0%)	*1/2 (50.0%)
Anti-HBe+	0/3 (0.0%)	0/3 (0.0%)	0/0	*1/7 (14.3%)	0/2 (0.0%)	*1/5 (20.0%)	3/10 (30.0%)	1/4 (25.0%)	2/6 (33.3%)	0/3 (0.0%)	*0/1 (0.0%)	*0/2 (0.0%)
Anti-HBs+ only	15/36 (41.7%)	1/13 (7.7%)	14/23 (60.9%)	14/60 (23.3%)	1/12 (8.3%)	13/48 (27.1%)	2/58 (3.5%)	2/23 (8.7%)	0/35 (0.0%)	0/60 (0.0%)	0/14 (0.0%)	0/46 (0.0%)
Anti-HBc+ total	1/36 (2.8)	0/13 (0.0%)	1/23 (4.4%)	0/60 (0.0%)	0/12 (0.0%)	0/48 (0.0%)	5/58 (8.6%)	1/23 (4.4%)	4/35 (11.4%)	7/60 (11.7%)	2/14 (14.3%)	5/46 (10.9%)
HBV naive	17/36 (47.2%)	9/13 (69.2%)	8/23 (34.8%)	38/60 (63.3%)	9/12 (75.0%)	29/48 (60.4%)	41/58 (70.7%)	16/23 (69.6%)	25/35 (71.4%)	45/60 (75.0%)	10/14 (71.4%)	35/46 (76.1%)
HCV+	0/36 (0.0%)			0/60 (0.0%)			4/58 (6.9%)			3/60 (5.0%)		
HDV+	0/3 (0.0%)			0/8 (0.0%)			2/10 (20.0%)			0/8 (0.0%)		

Data is indicated in numbers (%); HBsAg=hepatitis B surface antigen; HBeAg=hepatitis B e antigen; anti-HBe=hepatitis B e antibodies; HBV=hepatitis B; HCV=hepatitis C; HDV=hepatitis delta antigen, note: the proportions between HIV+ and HIV- do not correspond to HIV prevalence for both paediatric and adult cohorts, as samples were selected purposively by HIV status, the proportions between HBsAg+ and HBsAg- do not correspond to prevalence of HBsAg, as adult samples were selected purposively by HBsAg status, especially in the paediatric group sample volumes were limited, so that not all serological analyses could be performed; * are indicating the denominators that are smaller than in the overall group

**Hepatitis C virus:** seven of the 214 included paediatric samples (3.3%) tested positive for HCV antibodies (for age group stratified prevalences (Table 2)).

**Hepatitis D virus:** two of 185 paediatric samples (1.1%) tested positive for HDV antibodies (for age group stratified prevalences (Table 2)).

**Adult cohort:** for the adult cohort of the larger CODE study, comprising 2,349 individuals, the age distribution is left-censored due to a minimum age of 18 years as inclusion criterion. Individuals were categorized into the age groups 18 to 24, 25 to 29, and above 30 years of age. The maximum age was 46 years. The HBV and HIV-1 infection status of participants was determined at inclusion in the CODE study.

**Hepatitis B virus:** the prevalence of HBV infection was 5.0% (118/2,349), with comparable prevalence rates across the different adult age groups. The HIV-1 prevalence was 17.1% (402/2,349), with a significant increase in prevalence with increasing age (p<0.05). HIV infected adult individuals had a significantly higher prevalence of HBs antigen positivity (30/402, 7.5%) than HIV negative individuals (88/1947, 4.5%; χ^2^p<0.05). In the sub-cohort selected for the serological marker study, males represented 68/174 (39.1%) of the adult cohort.

In the subgroup of HBs-antigen negative adults, the frequency of total anti-HBc-antibody positivity increased with age, from 57.7% (30/52) in the age group 18 to <25 years up to 85.7% (42/49) in the age group >30 years. On the other hand, the proportion of HBV naive individuals decreased from 38.5% (20/52) to 12.2% (6/49), respectively (p<0.05, Table 1and Figure 2). Within the adult cohort, seemingly in contrast to the paediatric cohort, the HBe status showed comparable proportions of anti-HBe positivity in HIV negative (25/36, 69.4%) and HIV positive individuals (7/9, 77.8%) (Table 2).

**Hepatitis C virus:** two of the 174 included adult samples (1.1%) tested positive for HCV antibodies (for age group stratified prevalences (Table 3)).

**Table 3:** hepatitis B, C and D outcomes for selected subsets from the CODE adult cohort (2004)

	Adult cohort								
Age groups (years)	18 to <25			25 to <30			≥30		
	Overall N=75	HIV+ N=21	HIV- N=54	Overall N=35	HIV+ N=20	HIV- N=15	Overall N=64	HIV+ N=38	HIV- N=26
Male gender	30/75 (40.0%)	5/21 (23.8%)	25/54 (46.3%)	11/35 (31.4%)	4/20 (20.0%)	7(15 (46.7%)	27/64 (42.2%)	17/38 (44.7%)	10/26 (38.5%)
HBsAg+	21/75	2/21	19/54	7/35	3/20	4/15	14/64	4/38	10/26
HBeAg+	6/21 (28.6%)	1/2 (50.0%)	5/19 (26.3%)	2/7 (28.6%)	1/3 (33.3%)	1/4 (25.0%)	2/14 (14.3%)	0/4 (0.0%)	2/10 (20.0%)
Anti-HBe+	15/21 (71.4%)	1/2 (50.0%)	14/19 (73.7%)	6/7 (85.7%)	2/3 (66.7%)	4/4 (100.0%)	10/14 (71.4%)	4/4 (100.0%)	6/10 (60.0%)
Anti-HBs+ only	2/75 (2.7%)	1/21 (4.8%)	1/54 (1.9%)	0/35 (0.0%)	0/20 (0.0%)	0/15 (0.0%)	1/64 (1.6%)	0/38 (0.0%)	1/26 (3.9%)
Anti-HBc+ total	30/75 (40.0%))	17/21 (81.0%)	13/54 (24.1%)	20/35 (57.1%)	15/20 (75.0%)	5/15 (33.3%)	42/64 (65.6%)	32/38 (84.2%)	10/26 (38.5%)
HBV naive	20/75 (26.7%)	1/21 (4.8%)	19/54 (35.2%)	8/35 (22.9%)	2/20 (10.0%)	6/15 (40.0%)	6/64 (9.4%)	2/38 (5.3%)	4/26 (15.4%)
HCV+	1/75 (1.3%)			0			1/64 (1.6%)		
HDV+	0			0			1/64 (1.6%)		

Data is indicated in numbers (%); HBsAg=hepatitis B surface antigen; HBeAg=hepatitis B e antigen; anti-HBe= hepatitis B e antibodies; HBV=hepatitis B; HCV=hepatitis C; HDV=hepatitis delta antigen, note: the proportions between HIV+ and HIV- do not correspond to HIV prevalence, as samples were selected purposively by HIV status, the proportions between HBsAg+ and HBsAg- do not correspond to prevalence of HBsAg, as adult samples were selected purposively by HBsAg status; * are indicating the denominators that are smaller than in the overall group

**Hepatitis D virus:** one of the 174 included adult samples (0.6%) tested positive for HDV antibodies (for age group stratified prevalences (Table 3)).

## Discussion

This is the first systematic analysis on hepatitis B virus prevalence in Mbeya Region in Tanzania. The presence of two major trans-African highways and its relevance for sexually transmitted diseases emphasizes the importance of investigations on sexually transmitted infections in the area [24,29]. A high prevalence of HIV infections could be ascertained in the adult population, while only 5.0% were chronically infected with HBV, which corresponds to other reports from Africa [3]. However, more than half of the adult population showed serological signs of past infections with HBV, indicating a high exposure risk. Beyond the age of 30 more than 80% of adults had gone through an HBV infection in the course of their lifetime. This finding goes in line with reported low chronicity rates for HBV infections in adults, where most acute infections, which obviously continue to occur over the whole life-span within the naive population, spontaneously resolve. The presence of an HIV-1 infection is associated with an elevated risk of HBs antigen positivity [5]. Whether differences in risk behaviour lead to parallel increases in risk of HIV-1 and HBV infections, or whether a primary infection with either HIV-1 or HBV increases the odds of a chronic infection with the respective other infectious agent could not be resolved in this study. Increased odds of HBe-antigen positivity as an indicator of HBV replication could not be confirmed for HIV-1 co-infected individuals compared to HBV mono-infected individuals within the adult cohort.

In the paediatric cohort, an increasing prevalence of signs of exposure to HBV could be ascertained with increasing age. The prevalence of the constellation of HBs antigen negativity and total anti-HBc antibody positivity, which may be interpreted as resolved HBV infections, increased along the paediatric age groups. However, assumptions on life-time periods of increased exposure to HBV within the paediatric cohort have to be taken with caution in our study, as higher proportions of children could be observed in the age-groups from 0 to <7 years of age which showed a serological constellation that is indicative of previous vaccination. In general, the prevalence of HBs-antigen positivity was higher in children than in the adult group. In how far acute infections were represented through the revealed HBs-antigen positivity could not be elucidated with the available data. However, presence of acute infections in paediatric cases may explain the finding that in the adult population HBs-antigen positivity was found at lower frequencies; this can in turn be explained by clearance of infection or a die-off effect as the paediatric subpopulation ages into adulthood. This hypothesis is corroborated by previous reports of a higher probability of developing chronicity upon an acute infection in children than in adult patients [10,11**]**.

Systematic differences between the paediatric and the adult cohort, such as the time period of study inclusion, which was about 5 years later in the paediatric than in the adult cohort, may be one other confounding reason for differences between children and adults and remain one limitation of this study. In the younger age groups (0 to <8 years), a large proportion of individuals with anti-HBs antibodies only were identified, whereas this was practically absent in the older paediatric age groups (8 to <19 years) and in adults in general. It can be assumed that most of these children had been vaccinated against HBV. The authors are aware that such campaigns were conducted in childhood populations in the recent past prior to the study period, however, information both on population coverage of these campaigns and on individual vaccination status was not available for the corresponding time period. Our finding suggests a vaccination coverage of about 34% in the age group 0 to <8 years of age at the time of study. The finding is challenging intentions by the World Health Assembly to reduce new viral hepatitis B virus infections by 95% by 2030 as much higher levels of vaccination coverage will be needed to attain this goal [30]. The action framework on viral hepatitis by the WHO Regional Office for Africa states a 90% hepatitis B virus vaccine coverage for infants as target for 2020 [31]. To date pertinent information on hepatitis B vaccination coverage in the study area is not available.

A substantial limitation from this study is the heterogeneity of the two study cohorts: the EMINI cohort for the paediatric sub-cohort and the CODE cohort for the adult sub-cohort. Samples of the two sub-cohorts were collected at different time points and the diagnostic algorithms differ between the two cohorts. The analysis of HBs-antigen status was executed right at the time of inclusion as part of the original study protocol for the adults in the CODE study, whereas the HBs-antigen status in the paediatric EMINI participants was only executed several years after collection on frozen samples. In addition, the sero-status subgroups that were investigated in our study here were eventually becoming smaller as more serological criteria were added to the definition of subgroups, such as HBe sero-status. Studies that intend to provide more power in investigations on more complex constellations of HBV sero-status will have to draw from larger cohorts. In addition, the assessment of HBV or vaccine exposure status in participants if based on mere antibody- and antigen-constellations implies a certain degree of imprecision, as for example smaller parts of the anti-HBs only constellations may also be explained by the natural course of infection, as the use of isolated anti-HBs positivity as a sign of past vaccination is only indicative; similar concerns apply for HBs-antigen carriership as indication of chronic HBV infection, here for example occult infections cannot be ruled out as these can only be detected by systematic nucleic amplification techniques.

## Conclusion

Our data demonstrate a high prevalence of HBV infection in children in the Mbeya Region, with a large proportion appearing to clear the infection with increasing age. The exposure to HBV continues into adulthood, with ultimately more than 80% of adults beyond the age of 30 having undergone an HBV infection in the course of their lifetime. HBV therefore remains an understudied and underserved disease in the studied region in East Africa. Tackling this challenge will require combined efforts in the fields of prevention and effective disease management.

### What is known about this topic

- Although hepatitis B virus (HBV) infection is highly prevalent in sub-Saharan Africa available surveillance data is patchy;
- The infection is a frequent cause of morbidity and mortality due to liver function failure and malignancies;
- Management options in low resource settings are limited.

### What this study adds

- This study adds epidemiological data on hepatitis B virus (HBV) infection, along with data on HCV, HDV and HIV co-infection for a large region in Tanzania;
- The study allows to draw conclusions on the scope of existing vaccination strategies;
- The study supports stakeholders in designing adequate prevention and management strategies.
